# Mapping Verb Retrieval With nTMS: The Role of Transitivity

**DOI:** 10.3389/fnhum.2021.719461

**Published:** 2021-09-01

**Authors:** Effrosyni Ntemou, Ann-Katrin Ohlerth, Sebastian Ille, Sandro M. Krieg, Roelien Bastiaanse, Adrià Rofes

**Affiliations:** ^1^International Doctorate in Experimental Approaches to Language and Brain (IDEALAB, Universities of Groningen, Potsdam, Newcastle, Trento and Macquarie University), Sydney, NSW, Australia; ^2^Centre for Language and Cognition Groningen (CLCG), University of Groningen, Groningen, Netherlands; ^3^Department of Neurosurgery, Klinikum Rechts der Isar, School of Medicine, Technical University of Munich, Munich, Germany; ^4^Center for Language and Brain, National Research University Higher School of Economics, Moscow, Russia

**Keywords:** action naming, navigated transcranial magnetic stimulation, transitive vs. intransitive, parietal lobe, language mapping, argument structure

## Abstract

Navigated Transcranial Magnetic Stimulation (nTMS) is used to understand the cortical organization of language in preparation for the surgical removal of a brain tumor. Action naming with finite verbs can be employed for that purpose, providing additional information to object naming. However, little research has focused on the properties of the verbs that are used in action naming tasks, such as their status as transitive (taking an object; e.g., *to read*) or intransitive (not taking an object; e.g., *to wink*). Previous neuroimaging data show higher activation for transitive compared to intransitive verbs in posterior perisylvian regions bilaterally. In the present study, we employed nTMS and production of finite verbs to investigate the cortical underpinnings of transitivity. Twenty neurologically healthy native speakers of German participated in the study. They underwent language mapping in both hemispheres with nTMS. The action naming task with finite verbs consisted of transitive (e.g., *The man reads the book*) and intransitive verbs (e.g., *The woman winks*) and was controlled for relevant psycholinguistic variables. Errors were classified in four different error categories (i.e., non-linguistic errors, grammatical errors, lexico-semantic errors and, errors at the sound level) and were analyzed quantitatively. We found more nTMS-positive points in the left hemisphere, particularly in the left parietal lobe for the production of transitive compared to intransitive verbs. These positive points most commonly corresponded to lexico-semantic errors. Our findings are in line with previous aphasia and neuroimaging studies, suggesting that a more widespread network is used for the production of verbs with a larger number of arguments (i.e., transitives). The higher number of lexico-semantic errors with transitive compared to intransitive verbs in the left parietal lobe supports previous claims for the role of left posterior areas in the retrieval of argument structure information.

## Introduction

Navigated Transcranial Magnetic Stimulation (nTMS) is used to delimit (i.e., map) the cortical representation of language in preparation for the removal of a brain tumor in the context of an awake surgery (Tarapore et al., 2013; Babajani-Feremi et al., 2016; Freyschlag et al., 2018). This method can be administered preoperatively. Hence, it is possible to run language assessments unencumbered by issues that may accrue during surgery, such as poor compliance due to patient discomfort, interference with anesthetics, or problems correlating data from other preoperative assessments with intraoperative data due to brain shift (e.g., Santini et al., 2012; Adapa et al., 2014; Gerard et al., 2017). Unlike functional Magnetic Resonance Imaging (fMRI), the nTMS methodology mimics intraoperative mapping with Direct Electrical Stimulation (DES) by inducing errors during word production tasks. In these errors lies the value of preoperative nTMS. Areas that induce errors when stimulated (i.e., nTMS-positive areas) are considered to support functions necessary to perform the given language task (Pascual-Leone, 2000; Hartwigsen et al., 2013; Genon et al., 2018).

At the clinical level, the presence of errors during nTMS is used to guide the surgical procedure by issuing recommendations on the basis of points that elicit a positive or negative reaction (Picht et al., 2013; Krieg et al., 2017; Freyschlag et al., 2018). At a more theoretical level, nTMS-induced errors can be used to point to the function(s) affected during stimulation and, hence, enhance our understanding of the cortical organization of language (Corina et al., 2010; Moritz-Gasser et al., 2013; Sarubbo et al., 2015). For example, anomias and semantic paraphasias may emerge due to the inhibition of the lexico-semantic system, whereas word fragments or phonemic approximations may appear due to inhibiting phonological or articulatory processes (Moritz-Gasser et al., 2013; Picht et al., 2013; Rofes and Miceli, 2014; Hauck et al., 2015; Rofes et al., 2019).

Object naming has been used in nTMS studies to examine the representation of language in the brain (Picht et al., 2013; Hauck et al., 2015; Ille et al., 2015; Krieg et al., 2017). During object naming, participants name black and white drawings of objects and animals. This task engages the general storage of meaning (i.e., the semantic system), as well as the retrieval and production of nouns (i.e., lexical and articulatory processes). However, object naming does not engage all language processes that are necessary to build sentences. For that, verbs are needed: they are used for reference to an event and they include information about argument structure and thematic roles, necessary features to build a sentence (Rofes and Miceli, 2014; Rofes et al., 2015; Bastiaanse et al., 2016). These features are relevant for everyday communication and can be easily assessed with another task, that is, action naming.

The design and administration of action naming tasks is similar to object naming tasks. In action naming tasks, participants are shown black and white drawings of a character or animal carrying out an action, and participants are asked to name the event using an infinitive *(to wink)*, gerund *(winking)*, or to produce the subject along with the verb in the correct inflected form *(she winks)*. Several studies using nTMS have contrasted object and action naming (Hernandez-Pavon et al., 2014; Hauck et al., 2015). Hauck et al. (2015) reported a higher number of errors with action naming with infinitives compared to object naming only in posterior regions, whereas Ohlerth et al. (submitted) found overall more errors with action naming with finite verbs compared to object naming. The question that arises is whether linguistic variables of verbs (e.g., age of acquisition, regularity, transitivity, etc.) can affect the number of errors evoked by nTMS during action naming.

In this study, we explored a new approach to study nTMS data. We analyzed the types of verbs in an action naming task with a specific focus on the number of arguments that verbs can take (i.e., transitivity), specifically the difference between transitive (e.g., *he reads*) and intransitive verbs (e.g., *she winks*).

### Theoretical Background and Evidence From Individuals With Aphasia

Verbs differ in terms of their syntactic properties regarding the number and type of syntactic complements they take (e.g., Chomsky, 1993). For example, consider the verbs *to wink* and *to read* and the fact that they are different in terms of transitivity. *To wink* can express an event which involves only one entity, which has the thematic role of *agent* as shown in (1a). This type of verb is an *intransitive* verb (Grimshaw, 1990). The verb *to read* in (1b) expresses an event which includes two arguments, namely the agent (i.e., *Mary*) and the theme (i.e., *a book*). The difference between intransitive and transitive verbs becomes obvious when we consider (1c) and the fact that such an utterance does not obey grammatical rules (i.e., it is ungrammatical). However, a missing object does not render (1d) ungrammatical, because *to read* is a *pseudo-transitive* verb. In this respect they differ from obligatory transitive verbs, such as *to fix* that require an object (1e vs. 1f). Pseudo-transitive verbs (e.g., *to eat, to read, etc*.) still require two arguments, even if the object is not phonologically/overtly produced (Rappaport Hovav and Levin, 2002; Levin, 2006). According to Rappaport Hovav and Levin (2002), the reason behind object dropping is that the patient/theme of pseudo-transitive verbs is part of their meaning. As a result, their object, which is a grammatical entity, is not always required to be produced.

(1) a. Mary is winking.
b. Mary is reading a book.
c. ^*^Mary is winking a book.
d. Mary is reading.
e. ^*^Mary is fixing.
f. Mary is fixing a bicycle.

Different cognitive models account for the influence of transitivity in single word and sentence production. Lexicalist approaches argue that grammatical information is stored within the lemma (i.e., syntactic properties and meaning of a word; Roelofs et al., 1998) and is retrieved regardless of the presence of sentence context (Bock and Levelt, 1994; Levelt et al., 1999; Bastiaanse and van Zonneveld, 2004; Bastiaanse et al., 2016). This presupposes that retrieval of verbs with more complex argument structures are more costly because of the increased complexity of the lemma (Bock and Levelt, 1994; Levelt et al., 1999; Bastiaanse and van Zonneveld, 2004; Bastiaanse et al., 2016).^1^ However, weak lexicalist theories state that grammatical information is only retrieved given the presence of sentence context (Caramazza, 1997), while constructivist accounts argue that grammatical information is not associated with lexical entries and that sentence building is restricted by semantics and world knowledge (Borer, 2005). These are relevant points for the present study because our action naming task requires the verb to be retrieved in sentence context (Ohlerth et al., 2020). As a result, for our task all accounts predict that producing a transitive verb in sentence context is more complex than producing an intransitive verb in sentence context because grammatical information has to be accessed and encoded when constructing a sentence. Retrieval and encoding of argument structure information takes place regardless of whether arguments are phonologically/overtly expressed (Bastiaanse and van Zonneveld, 2004; Thompson et al., 2007; den Ouden et al., 2009).

Predictions of theoretical frameworks regarding transitivity have been confirmed by experimental evidence from individuals with agrammatic aphasia. In spontaneous speech, people with post-stroke agrammatic aphasia and Alzheimer's disease tend to produce relatively more intransitive verbs and fewer transitive verbs than non-brain damaged speakers (Bastiaanse and Jonkers, 1998; Kim and Thompson, 2004). However, the same does not hold true for action naming, as only subgroups of individuals with agrammatic aphasia face difficulties producing the finite form of transitive verbs (Jonkers, 2000), and while results may hold at the group level, this is not always the case at the individual level (Luzzatti et al., 2002; De Bleser and Kauschke, 2003). Cho-Reyes and Thompson (2012) also showed that people with agrammatic aphasia showed difficulty producing both pseudo- and obligatory transitive verbs in a sentence context (e.g., *to read*) compared to intransitive verbs.

In sum, reports on people with agrammatic aphasia indicate more difficulties with transitive than intransitive verbs either in spontaneous speech or action naming tasks. Some of the aforementioned studies indicate the location of neurological damage, typically in perisylvian areas of the left hemisphere (i.e., Jonkers, 2000; Kim and Thompson, 2004; cf. Bastiaanse and Jonkers, 1998; Luzzatti et al., 2002; De Bleser and Kauschke, 2003). However, the level of brain damage due to stroke or neurodegeneration is commonly too large to pinpoint specific brain areas involved in the processing of argument structure. Hence, a close look at the neuroimaging literature seems relevant.

### Evidence From Neuroimaging

fMRI studies have examined the influence of argument structure during sentence and single word comprehension, as well as single word production. Evidence from sentence comprehension connects left posterior temporal and inferior frontal regions with the processing of verbs that assign an increased number of arguments and thematic roles (Ben-Shachar et al., 2003; Shetreet et al., 2007; Malyutina and den Ouden, 2017).

In a series of studies with non-brain-damaged participants, Thompson and colleagues employed lexical decision tasks. The authors reported that the processing of transitive verbs generates higher activation in inferior parietal regions of the left and right hemisphere (i.e., angular and supramarginal gyrus) compared to the processing of intransitive verbs (Thompson et al., 2007, 2010). Due to the absence of sentence-context from the task (i.e., lexical decision), these results were interpreted as evidence for lexicalist accounts of argument structure.

Most research with fMRI has examined argument structure processing during comprehension. An exception is the study by den Ouden et al. (2009), in which the authors investigated argument structure in overt action naming conditions using pictures and videos. Across presentation modes, transitive verbs yielded activation in left and right areas of the posterior temporal lobe, inferior and superior parietal lobe, as well as left inferior frontal gyrus. Surprisingly, intransitive verbs also yielded more activation than transitives in precentral and middle temporal areas of the left as well as right hemisphere (den Ouden et al., 2009). Neuroimaging and aphasiological evidence comprised the basis for Thompson and Meltzer-Asscher (2014) neurocognitive model of argument structure, which considers argument structure information retrieval as function of the left and right inferior parietal lobes.

**Figure 1:**
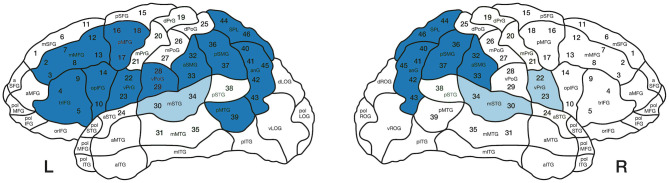
Summary of areas that have been reported to elicit more activation during the processing of transitive over intransitive verbs (blue areas) and those for the opposite contrast (i.e., more activation for intransitive compared to transitive verbs; light blue areas). Numbers indicate the 46 stimulation points used for the current study according to CPS regions (Corina et al., 2010). L, Left hemisphere; R, Right hemisphere.

TMS over the left inferior parietal lobe has also been shown to facilitate thematic role assignment, adding causal evidence to the role of posterior regions in argument structure processing (Finocchiaro et al., 2015; Vercesi et al., 2020). Hence, previous findings consistently show that more arguments engage bilateral temporoparietal regions during comprehension, whereas frontal regions can also be engaged during the production of transitive verbs (Ben-Shachar et al., 2003; Thompson et al., 2007, 2010; den Ouden et al., 2009; Meltzer-Asscher et al., 2015). Figure 1 summarizes the cortical regions reported to be more activated during the processing of transitive compared to intransitive verbs and the opposite.

### Aim of The Present Study

The aim of the present study is to explore the cortical representation of transitive and intransitive verbs with nTMS and to investigate whether transitivity affects the number and localization of nTMS-induced errors. Hence, we ask the following questions:

1. Does nTMS induce more (or fewer) errors with transitive compared to intransitive verbs?
2. If so, in which cortical regions (i.e., hemisphere and lobes) can we localize the nTMS-induced error rates for transitive and intransitive verbs?
3. Do transitive and intransitive verbs elicit different types of nTMS-induced errors?

Based on previous literature, we assumed that if transitive verbs generate larger cortical activity as seen in aforementioned neuroimaging studies, nTMS would induce more errors with transitive compared to intransitive verbs in posterior regions. Concerning specific error categories, we hypothesized that if the locus of complexity for transitive verbs compared to intransitive verbs is indeed the lexico-semantic level, then we will observe more errors of the lexico-semantic category.

## Methods

We analyzed previously reported data by focusing solely on the comparison between transitive and intransitive verbs during action naming under nTMS (Ohlerth et al., submitted).

### Participants

Twenty neurologically healthy participants were tested. They ranged in age 20–53 (mean age: 24.75, SD = 7). They were 12 females, 1 left-handed, and 1 ambidextrous individual. The inclusion criteria were: (1) German as a native language, (2) age of at least 18 years, (3) no contra-indications for Magnetic Resonance Imaging (MRI) 3 Tesla and/or nTMS mapping (i.e., use of cardiac pacemakers or devices for deep brain stimulation), (4) no neurological or psychiatric disorders, and (5) no pregnancy. Handedness was measured using the Edinburgh Handedness Inventory (Oldfield, 1971). The demographic data of the participants are given in Supplementary Table 1.

### MRI

Anatomical T1-weighted MRI images were acquired using a 3-Tesla magnetic resonance scanner (Achieva dStream; Philips Healthcare, Best, The Netherlands). 3D models of each participant's brain were constructed based on the acquired MRI images. These models were used for the guidance of coil placement during language mapping with stimulation (Nexstim eXimia NBS system version 4.3).

### Materials

**Figure 2:**
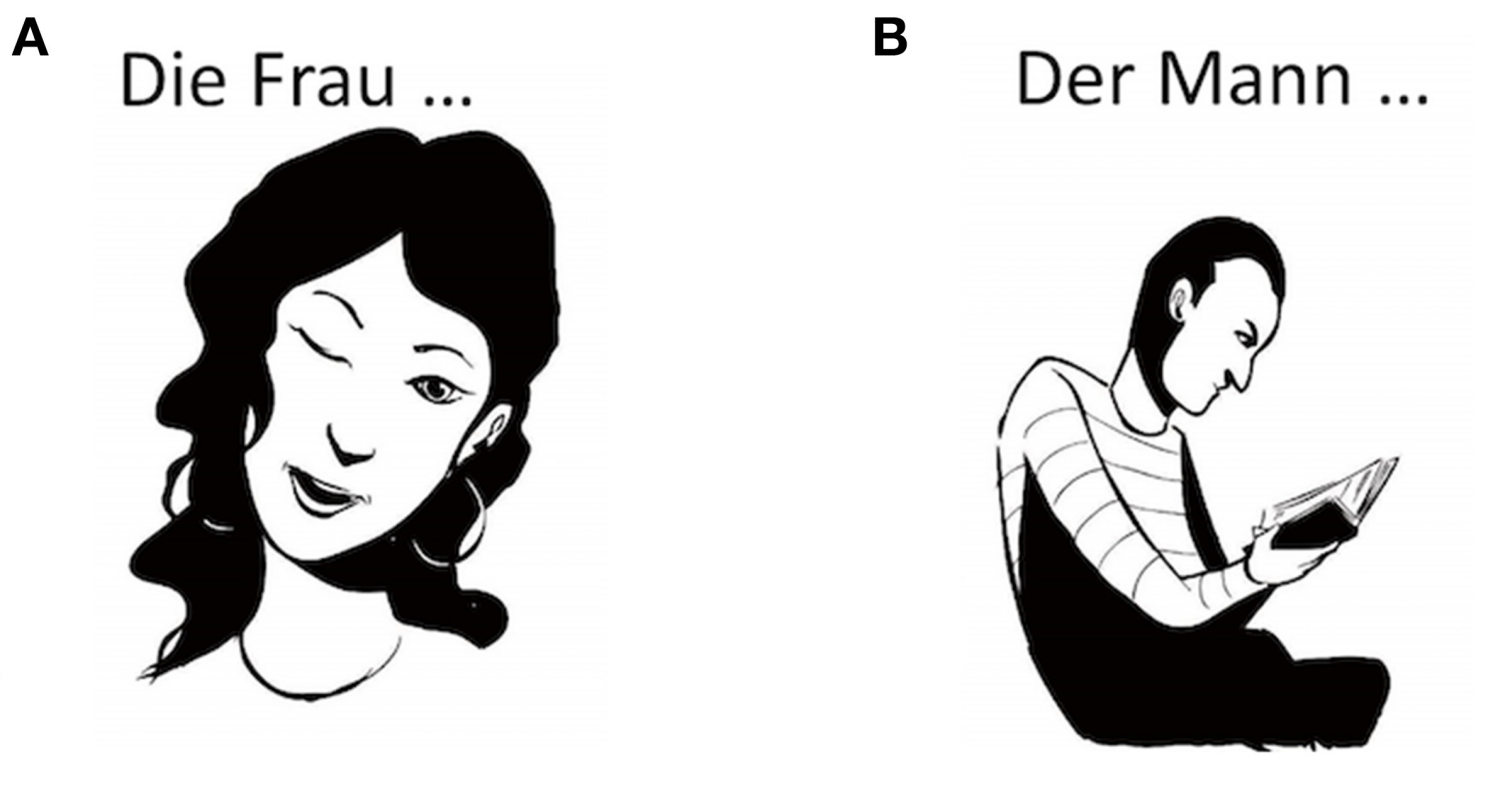
Example of an intransitive **(A)** and a transitive item **(B)** used during nTMS language mapping. The intransitive item triggered the sentence *Die Frau…zwinkert*: “The woman…winks,” while the transitive item triggered the sentence *Der Mann…liest*: “The man…reads”. Art work by Victor Xandri Antolin. © University of Groningen.

The German version of the Verb And Noun test for Peri OPerative testing (VAN-POP; Ohlerth et al., 2020) was used. For the current study, only the data of the verb test were included. The task consists of 75 black-and-white line drawings of actions, 22 of which corresponded to intransitive verbs and 53 to pseudo-transitive verbs (see Figure 2). Thirty-nine items for transitive verbs displayed an agent and a theme, 14 included the agent performing an action (e.g., Der Mann…kehrt; The man…sweeps). A lead-in phrase on top of each image provides the sentence context and triggers inflection for person, number, and tense (*Die Frau…zwinker****t***_3*rdpersonsingular, present*_*:* “The woman…wink**s**;” see Figure 2), which is the most natural way to use a verb. All items were balanced for factors known to affect naming performance such as word frequency, age of acquisition, length in syllables, regularity, instrumentality, and name-relatedness to the noun (Martin et al., 1989; Bastiaanse et al., 2016; Ohlerth et al., 2020). To confirm that our stimuli did not differ in terms of image complexity, we conducted an online survey with 25 native German speakers, who rated our stimuli on a 5-point Likert scale. These ratings showed that image complexity between transitive and intransitive verbs did not differ. To calculate whether frequency, age of acquisition, and naming agreement differed, we used the values provided by the VAN-POP (Ohlerth et al., 2020). Image complexity, frequency, age of acquisition, and naming agreement did not differ for any of our participants (see Supplementary Table 2).

### Procedure

#### Set-Up

A focal figure-of-eight coil was used. It produced biphasic pulses (length 23 mm) with maximal electric field strength of 172 V/m ± 2%. Prior to mapping, T-1 weighted MRI sequences were uploaded to the Nexstim eXimia NBS system version 4.3. According to the Cortical Parcellation System (CPS; Corina et al., 2010), 46 stimulation points were assigned to the 3D model of each participant and each stimulation point was allocated a number (see Figure 1). The cortical brain areas were visualized in relation to the orientation and focal point of the coil.

Surface electrodes for the recording of Motor Evoked Potentials were placed over the abductor pollicis brevis and abductor digiti minimi muscles to establish the Resting Motor Threshold (rMT) according to the preoperative language mapping protocol by Krieg et al. (2017). The TMS coil delivered single pulse stimulations over the motor cortex of the anatomical hand knob area to identify the most excitable spot. The ideal threshold that reproduced 5 out of 10 muscle movements higher than 50 μV was calculated and was used as the intensity for nTMS language mapping. The rMT was calculated for each hemisphere separately.

#### Baseline Testing

Participants were presented with the set of images one-by-one while seated ~60 cm in front of a computer screen. Picture presentation time (PPT) was set at 1,000 ms and inter-picture interval (IPI) at 2,500 ms. Participants were instructed to name the pictures as fast and accurately as possible. They were asked to overtly produce the lead-in phrase and the verb, while refraining from producing the object of transitive verbs (i.e., *The man…reads* instead of *The man. reads a book*, see Figure 2). This was done so that their answers would remain as short as possible. In that regard it is worth stressing that, given that we only included pseudo-transitive verbs, omitting the object did not result in ungrammatical sentences (see also section Theoretical Background and Evidence From Individuals With Aphasia; Ohlerth et al., 2020). In fact, when instructed to name the actions during baseline, participants hardly ever produced the object (<3%). In the rare occasions that participants did produce objects [e.g., *Der Mann isst (einen Apfel)* – The man is eating (an apple)], this was not considered an error and participants were encouraged to keep naming in a way that felt natural to them.

For each participant, two rounds of baseline naming were completed with the absence of any stimulation and misnamed items were excluded from the nTMS experiment for that participant. Hence, each person named an individualized set of images for the nTMS testing. This procedure is commonly used in preoperative language mapping (Krieg et al., 2017).

#### nTMS Mapping

The instructions for mapping with stimulation as well as IPI and PPT remained the same as during baseline naming. The interval between stimulation onset and picture presentation was set at 0 ms, so that picture and stimulation onset were synchronized. As it is common in language mapping protocols, we used repetitive stimulation with the intensity set at 5Hz/5 pulses, with a typical duration of 1,000 ms (Krieg et al., 2017). Stimulation intensity was set at 110% of the ipsilateral rMT for each hemisphere. Two rounds of nTMS stimulation were performed for each hemisphere and each round consisted of stimulating each CPS point (*N* = 46) a total of three times (i.e., 138 stimulations per round, 276 in total). Within each participant, the order of stimuli presentation was randomized and restarted once the list reached the end. The order of the stimulation of the hemispheres was balanced, so that half of the participants were first mapped on the left and half on the right hemisphere. Given our stimulation protocol, participants named each item 8 or 9 times, depending on the number of items excluded in the baseline. Mapping sessions were video and audio recorded.

#### Error Classification

A trained clinical linguist with expertise in nTMS language mapping performed all nTMS mappings and analyzed the *post-hoc* video recordings (A-K.O.). Video recordings of baseline naming were compared to recordings of naming under stimulation. Errors due to discomfort, pain, or stimulation of the peripheral facial nerves were excluded from the analysis. Building on previous literature on language mapping and in order to further summarize induced errors according to the level they point toward, the error classification is as follows (Corina et al., 2010; Picht et al., 2013; Rofes et al., 2017):

The category of *non-linguistic errors* includes *no responses* and *hesitations (on the sentence)*. No-response errors refer to a complete lack of intelligible response, including absence of the lead-in phrase. Hesitations (on the sentence) refer to delayed onset of the entire target sentence compared to the baseline naming. The category of *lexico-semantic errors* includes three different error types, namely *anomias, semantic paraphasias*, and *hesitations (on the target)*. Errors are classified as *anomias* when the lead-in phrase is correctly pronounced but the target is missing. Errors are classified as *semantic paraphasias* when the target word is replaced by a different existing word (e.g., *run* for *sleep*) and as *hesitations* (on the target) when the lead-in phrase is produced correctly, but the target word is delayed. The category of *grammatical errors* includes the wrong inflection of the lead-in phrase or the target (e.g., ^*^Die Frau.laufe: “The woman.run_**1stpersonsingular**_”). Last, the category of *errors at the sound level* includes two error types, *performance errors* and *phonological paraphasias*. Performance errors comprise stuttered or slurred speech (e.g., *Die Frau.s-s-schläft:* “The woman.s-s-sleeps”), while phonological paraphasias refer to substitution or omission of phonemes with the target word remaining recognizable (e.g., *Die Frau*.^*^täuft; “The woman.^*^tuns”).

#### Statistical Analyses

We conducted quantitative analysis of the errors as well as a qualitative analysis according to error types. Quantitatively, to check for differences between transitive and intransitive verbs, we conducted chi-square tests for each hemisphere and lobe. The induced errors were then divided into different error types and chi-square tests were conducted for each hemisphere and lobe, to see whether the quality of errors differed between transitive and intransitive verbs. Significance values were corrected for False Discovery Rate (i.e., FDR; Benjamini and Hochberg, 1995).

## Results

### Quantitative Analysis

nTMS stimulation induced errors in all participants. The mean number of items that were misnamed and, hence, excluded after baseline testing was 11.00 (sd = 4.4). The number of nTMS stimulations across participants was 11,040. Of these, 918 stimulations induced errors (8.3%). Errors were elicited in both hemispheres, with 429 errors occurring in the left hemisphere (3.9%) and 489 in the right (4.4%). A paired *t*-test revealed no significant difference between the number of errors per hemisphere (*t* = −1.15, *p* = 0.26).

Out of the 7,507 stimulations with transitive items, 661 stimulations elicited errors (8.8%). Out of the 3,533 stimulations with intransitive items, 257 stimulations induced errors (7.3%). This difference was significant (χ^2^ = 7.18, *p* =0.007).

**Figure 3:**
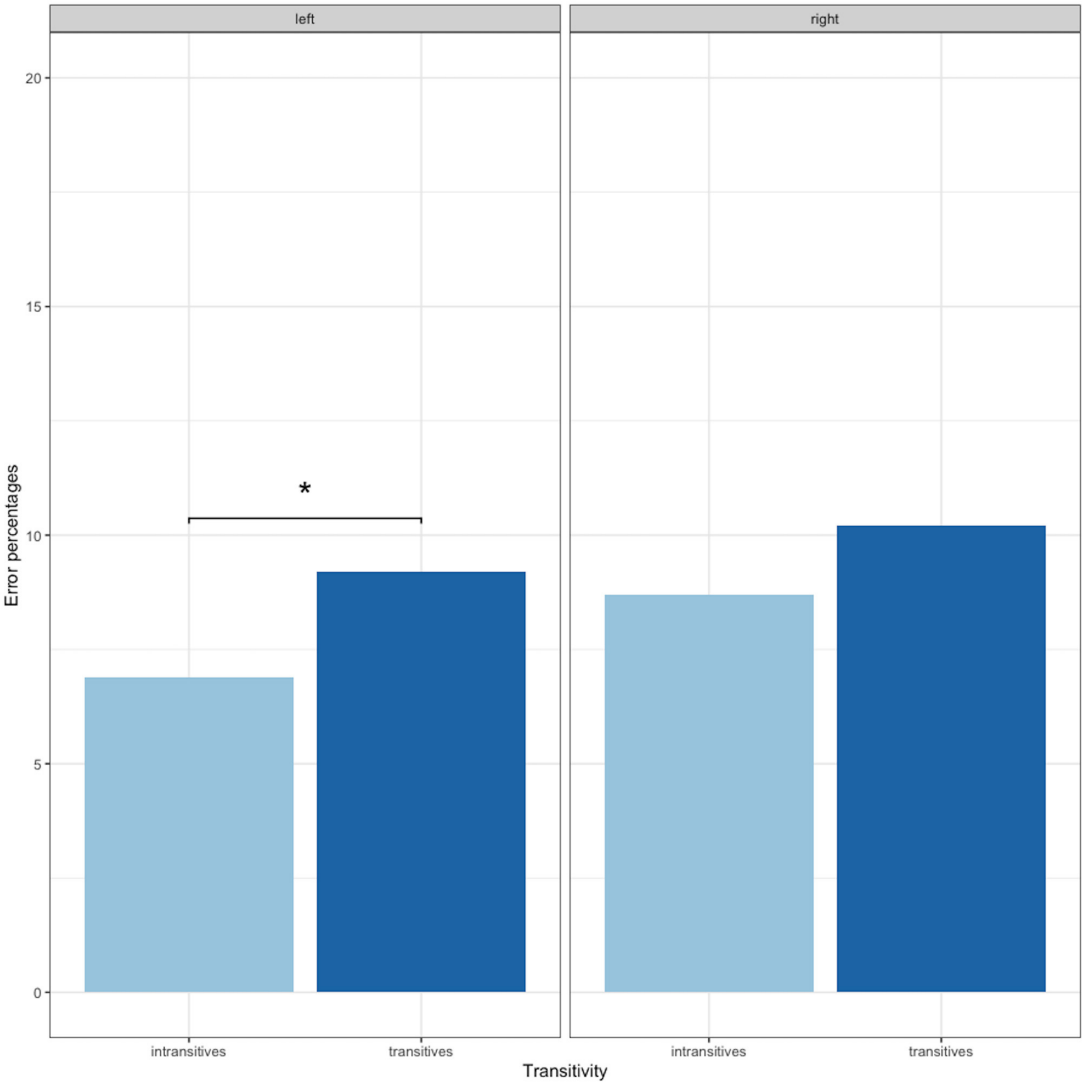
Error percentages of nTMS induced errors according to verb type and hemisphere. The axis has been set to 20% for purposes of visualization. ^*^p < 0.05.

Analyzing the effect of transitivity per hemisphere, significantly more nTMS-positive points were identified with transitive items in the left hemisphere (transitives = 9.2%; intransitives = 6.9%; χ^2^ = 5.49, *p* = 0.02 after FDR adjustment), while we found no differences in the right hemisphere (transitives = 10.2%; intransitives = 8.7%; χ^2^ = 2.00, *p* = 0.15; see Figure 3).

Regarding our lobe-wise analysis, a significantly higher number of nTMS-induced errors was elicited during the production of transitive verbs compared to intransitive verbs when stimulating the left parietal lobe (transitives = 9%; intransitives = 5.4%; χ^2^ = 6.28, *p* = 0.03 corrected for FDR). No differences were found in the right parietal lobe (transitives = 9.3%; intransitives = 6.8%; χ^2^ = 3.03, *p* = 0.08), nor in the temporal lobes (left: transitives = 8.6%; intransitives = 7.8%; χ^2^ = 0.06, *p* = 0.80; right: transitives = 9.4%; intransitives = 7.4%; χ^2^ = 0.69, *p* = 0.40) and in the frontal lobes (left: transitives = 7.9%; intransitives = 6.8%; χ^2^ = 0.87, *p* = 0.35; right: transitives = 9%; intransitives = 8.8%; χ^2^ = 0.005, *p* = 0.94).

### Qualitative Analysis

The most frequently induced error category was lexico-semantic errors that accounted for 45.8% of all errors (*N* = 421), followed by errors at the sound level with 30.5% (*N* = 280), non-linguistic errors with 18.6% (*N* = 171), and grammatical errors with 5% (*N* = 46). Table 1 shows percentages of the subdivisions that comprised each error category across both hemispheres and according to verb type.

**Table 1:** Results of the qualitative analysis across hemispheres (percentages of errors).

**Error category**	**Subdivision**	**Verb type**	**Percentage**
Non-linguistic errors	Hesitation on the sentence	Transitive Intransitive	16.04 17.12
	No response	Transitive Intransitive	2.12 2.72
Lexico-semantic errors	Hesitation on the target	Transitive Intransitive	33.89 31.52
	Anomia	Transitive Intransitive	5.45 5.45
	Semantic	Transitive Intransitive	6.81 8.16
Grammatical errors	Grammatical	Transitive Intransitive	4.84 5.44
Sound level	Phonological	Transitive Intransitive	0.30 0.00
	Performance	Transitive Intransitive	30.56 29.60

In the left hemisphere, there was no difference between the error categories for the transitive nor the intransitive verbs. When examining the separate lobes of the left hemisphere, no significant differences were found in the frontal and temporal lobes, but in the left parietal lobe, we found significantly more nTMS-induced lexico-semantic errors with transitive than with intransitive verbs (4.6 vs. 2.2%). A summary of the results according to error category and lobe can be found in Table 2.

**Table 2:** Error types elicited in the left hemisphere per lobe.

**Lobe**	**Non-linguistic errors**	**Lexico-semantic errors**	**Grammatical errors**	**Sound level**
Frontal	χ^2^ =0.31, *p* = 0.57	χ^2^ = 0.00, *p* = 0.99	W = 0.35, *N* = 0.00, *p* = 0.75	χ^2^ = 0.50, *p* = 0.47
Temporal	W = 0.28, *N* = 0.00, *p* = 0.83	χ^2^ = 0.00, *p* = 1	–	W = −1.2, *N* = 0.99, *p* = 0.29
Parietal	χ^2^ = 0.11, *p* = 0.73	χ^2^ = 5.38, *p* = 0.04^*^	–	χ^2^ = 0.45, *p* = 0.50

*–, no errors of this type occurred; W, Wald statistic; N, Nuisance parameter;*

* *p-value after FDRcorrection*.

No significant differences were found between the error types for transitive and intransitive verbs in the right hemisphere (see Supplementary Table 3). No error category appeared to be more frequent with transitive than with intransitive items in the right frontal, temporal, or parietal lobe (see Supplementary Table 4).

## Discussion

In the present study, nTMS induced a larger number of errors with transitive compared to intransitive verbs. Examining each hemisphere separately, the error rate with transitive verbs was higher than that of intransitive verbs in the left hemisphere and particularly in the left parietal lobe. Qualitatively, more lexico-semantic errors with transitive items compared to intransitive items were produced during stimulation in the left parietal lobe. These findings will be discussed within the above-presented theoretical context and evidence from behavioral studies in people with aphasia, as well as in relation to neuroimaging studies.

### Linguistic Theories and Evidence From Aphasia

Theories of argument structure predict that the complexity of transitive verbs is associated with linguistic processes either at the lemma or at the sentence level (Bock and Levelt, 1994; Caramazza, 1997; Levelt et al., 1999; Hale and Keyser, 2002; Borer, 2005; Marantz, 2013). Regardless of the specific processing levels, the production of finite transitive verbs in sentence context requires more complex lexico-semantic and syntactic processes than the production of intransitive verbs. Our data confirm this: more nTMS-induced errors were found with transitive verbs in the left hemisphere, while in the right hemisphere, this effect was not observed. The left hemisphere has traditionally been linked to language processing and is considered the language-dominant hemisphere (Knecht et al., 2000; cf. see Hartwigsen et al., 2010a,b for the contribution of right hemisphere regions in phonological processing). Thus, the presence of more nTMS-positive points with transitive items in the left but not in the right hemisphere indicates that the error rates of the two verb types are affected by linguistic factors, in this case transitivity.

Regarding the exact linguistic features that render transitive verbs more complex, the present study cannot offer resolution. As previously stated, we opted for an action-naming-in-sentence-context task to elicit verbs inflected for number, person, and tense (e.g., *The woman.winks*). Hence, our experimental design does not allow to draw conclusions in terms of whether argument structure information is stored at the level of lexical entries, because all models agree with the fact that verbs in sentence context engage grammatical information.

Previous studies that implemented sentence context in action naming either presented sentences with varying number of arguments (e.g., Ben-Shachar et al., 2003; Shetreet et al., 2007) or asked participants with aphasia to produce the verb and its corresponding direct object (e.g., Jonkers, 2000). Our study used a naming task providing a consistent sentence context. That is, we used an action naming task with a lead-in phrase that included a determiner and a noun (i.e., *The man/The woman*) which was kept the same across conditions (i.e., transitives/intransitives). Based on our results, we argue that even when sentence context is kept constant and the second argument is not overtly expressed, transitive verbs require a larger amount of argument structural information to be activated compared to intransitive verbs. According to lexicalist approaches, information about argument structure is retrieved with the lemma regardless of the presence of sentence context (Bock and Levelt, 1994; Levelt et al., 1999; Bastiaanse and van Zonneveld, 2004; Kim and Thompson, 2004; Thompson et al., 2007; Bastiaanse et al., 2016). Hence, since transitive verbs carry more arguments (i.e., agent + theme), their retrieval is more challenging compared to intransitive verbs (i.e., agent) and therefore, more easily disturbed by nTMS inhibition.

However, the locus of increased complexity of transitive verbs compared to intransitives is not necessarily exclusively due to lemma retrieval. As previous studies on post-stroke aphasia have demonstrated, observed difficulties with verb production are due to impairments affecting the level of *grammatical encoding* (Bock and Levelt, 1994; Levelt et al., 1999; Bastiaanse and van Zonneveld, 2004). According to this view, verbs with more complex argument structure need to grammatically encode more information (i.e., transitives) compared to verbs with simple argument structure (i.e., intransitives). This has been shown to affect finite (Jonkers, 2000; Bastiaanse and van Zonneveld, 2004, 2005) as well as non-finite verb production (Luzzatti et al., 2002; Kim and Thompson, 2004). Hence, an increased number of nTMS-induced errors for transitive compared to intransitive verbs is not only attributable to lemma retrieval, but also to the grammatical encoding of retrieved lemma level information.

### The Role of The Left Parietal Lobe

Our findings stress the role of the left parietal lobe regarding argument structure. Parietal areas reported in previous neuroimaging studies were argued to function as a repository of information regarding argument structure (Thompson et al., 2007, 2010; den Ouden et al., 2009; Thompson and Meltzer-Asscher, 2014; Meltzer-Asscher et al., 2015). Cortical activation of these areas is higher when processing transitive compared to intransitive verbs. This is because the amount of argument structure information associated with transitive verbs is higher (Thompson et al., 2007, 2010; den Ouden et al., 2009; Thompson and Meltzer-Asscher, 2014; Meltzer-Asscher et al., 2015).

In our study, nTMS induced more lexico-semantic errors with transitive items in the left parietal lobe. These errors are associated with the level of access to the lexicon (Corina et al., 2010; Bastiaanse et al., 2016; Rofes et al., 2017). If left parietal areas serve as a repository of argument structural information, as has been previously argued (Thompson et al., 2007; den Ouden et al., 2009; Thompson and Meltzer-Asscher, 2014), then inhibition of these areas results in difficulty accessing lexico-semantic information of the verb. Hence, the larger number of lexico-semantic errors in the current study adds causal evidence to the function of the left parietal lobe in argument structure information retrieval, as previously suggested by fMRI data (i.e., Thompson et al., 2007, 2010; den Ouden et al., 2009; Thompson and Meltzer-Asscher, 2014; Meltzer-Asscher et al., 2015).

Apart from argument structure information retrieval, the left parietal lobe has also been connected with grammar. Evidence from Basque and English shows that syntactic anomalies, such as case or verb agreement violations, activate the left and right inferior parietal lobes, whereas semantic anomalies do not (Kuperberg et al., 2003, 2008; Nieuwland et al., 2012). Syntactic judgments are not the only grammatical tasks that have generated increased activity in parietal areas. Naming finite verbs as well as sentence completion were also shown to activate the left and right inferior parietal cortex compared to simpler lexical tasks in a group of individuals with brain tumors (Połczyńska et al., 2017). Since transitive verbs need to encode more grammatical information compared to intransitive verbs (Levelt et al., 1999; Bastiaanse and van Zonneveld, 2004), they engage parietal areas to a greater extent. As a result, nTMS over these areas induces more errors with transitive verbs during our action naming task.

### The Role of The Right Parietal Lobe

Although previous literature reported increased activation with transitive compared to intransitive verbs in right posterior regions, we did not observe more nTMS-induced errors for transitive compared to intransitive verbs in the right hemisphere (Thompson et al., 2007; den Ouden et al., 2009). The reason behind the lack of such an effect is 2-fold.

First, previous studies have reported right hemisphere activation for experimental designs and conditions that differ from those in the present study. den Ouden et al. (2009) administered an action naming task without sentence context where participants were asked to produce non-inflected verbs (e.g., *run*). They used two presentation modes, static pictures and dynamic videos. The effects for transitive items within the video condition were more lateralized in right parietal regions than the effects for pictures (den Ouden et al., 2009). Thompson et al. (2007) also reported the involvement of regions in the right hemisphere for transitive, but not intransitive items. However, parietal regions were activated only for the contrast between ditransitive (e.g., *give*) and transitive verbs (e.g., *read*) vs. intransitives (e.g., *wink*). In our study, we did not include ditransitive items or dynamic videos as a presentation mode. Thus, right parietal areas may have been reported for specific conditions and experimental designs, which were not used in the present study.

Since Broca's findings on patient Tan (Broca, 1865), the notion that language dominance resides in the left hemisphere has prevailed in the field (cf. Labache et al., 2020). Early TMS studies have supported the traditional view on language lateralization. Pascual-Leone et al. (1991) examined whether TMS could determine language lateralization in individuals with epilepsy. They reported that TMS was able to reliably elicit speech arrests in the left but not in the right hemisphere, a result that led to the conclusion that TMS is able to detect left hemispheric language dominance (for a review on TMS studies and language dominance with a focus on the right hemisphere, see Hartwigsen and Siebner, 2012). Preoperative mapping protocols for nTMS have also been used for detection of language dominance, with studies reporting left hemisphere dominance for healthy volunteers but a functional language shift to the right hemisphere for individuals with brain tumors (Krieg et al., 2013; Ille et al., 2016). However, nTMS-induced errors in healthy volunteers have been reported to be bilaterally distributed, with performance errors and no responses being the most frequently induced error types in the right hemisphere (Sollmann et al., 2014). Since the main question of the present study was related to different verb types, we did not examine whether error categories differed according to hemisphere. For a more detailed discussion on right hemisphere contributions using the same protocol and action naming, please, see Ohlerth et al. (submitted) as well as Ohlerth et al. (this issue).

Within the context of transitivity, unlike previous literature that employed fMRI, non-correlational techniques, such as TMS, potentially affect necessary processes for verb production (Genon et al., 2018). Hence, right parietal regions may have a supportive role which cannot be significantly disrupted with nTMS. In our study, this is reflected in the lack of an increase of lexico-semantic errors with transitive verbs in right parietal areas. Even though previous work on language mapping with nTMS has reported contributions of right parietal areas in object and action naming, lexico-semantic errors do not appear to be more induced in right parietal areas compared to left (Krieg et al., 2013; Sollmann et al., 2014; Ohlerth et al., submitted).

### Clinical Implications

Our findings highlight the importance of administering linguistically motivated naming tasks within the context of preoperative nTMS language mapping. Specifically, concerning action naming tasks, we suggest that the variable “transitivity” should be controlled. Regardless of the reasons behind the increased lexico-semantic complexity of transitive verbs, it becomes apparent that transitivity affects the number and localization of nTMS-positive points. Careful consideration of these variables during the construction of action naming tasks is, hence, important for language mapping with nTMS.

In the present study, we reported that an increased amount of argument structure information particularly affects the left parietal lobe. This finding indicates that when clinicians map peritumoral regions in left parietal areas with nTMS, they may opt for an action naming task with transitive items or at least consider such items in the task at hand. The use of exclusively intransitive items may lead to low numbers of induced errors, that may lead to the conclusion that these areas are not involved in language. This is also emphasized by a recent review of TMS and DES language mapping, which reported that the highest number of false-negative TMS points is located in posterior language areas (Jeltema et al., 2021).

### Limitations and Future Directions

It should be noted that the exact number of stimulations per lobe with transitive and intransitive items was not available to the researchers. This is because the video analysis software of the present study is blinded to the stimulation site. Once an error has been identified, the stimulation sequence is stored and marked on the anatomical space. However, this does not apply to stimulations that did not induce errors. Hence, to conduct lobe-wise analyses, we assumed that the distribution of the presented transitive and intransitive items was equivalent to the overall distribution of our items (i.e., 68% transitive items and 32% intransitive). Our assumption is justified by the fact that the action naming task was presented 8-9 times per participant and was randomized every time after the final item. Several questions arise from our findings: Evidence from pre- and intraoperative studies with nTMS and DES in people with brain tumors can be used to cross-validate our findings and show whether similar effects can be found in individuals with neuro-pathologies. Additionally, it seems important to examine word properties of verbs (and nouns) within mapping with nTMS and DES. Large sets of nTMS data can be analyzed to identify word properties affecting naming accuracy and proneness to nTMS disruption (Alyahya et al., 2020). Furthermore, error types should be examined thoroughly in connection to locus of stimulation, task, and word properties. Future work should also investigate whether cortical differences between different verb types affect nTMS-guided tractography (Raffa et al., 2016; Negwer et al., 2017; Giampiccolo et al., 2020; Ohlerth et al., submitted).

## Conclusion

Our study shows that the number as well as type of nTMS-induced errors can be differentially affected based on the items of an action naming task. In particular, it provides causal evidence for the complexity of transitive verbs (vs. intransitives verbs) and their cortical representation in the left parietal lobe.

## Data Availability Statement

The raw data supporting the conclusions of this article will be made available by the authors, without undue reservation.

## Ethics Statement

The studies involving human participants were reviewed and approved by the Ethics Committee of the Technical University of Munich. The patients/participants provided their written informed consent to participate in this study.

## Author Contributions

EN, A-KO, SI, SK, RB, and AR: conceptualization, methodology, validation, investigation, and writing—review and editing. EN, A-KO, and AR: formal analysis. SI and SK: resources and funding acquisition. EN and A-KO: data curation. EN, A-KO, AR, and RB: writing—original draft preparation. EN: visualization. SI, SK, RB, and AR: supervision and project administration. All authors contributed to the article and approved the submitted version.

## Conflict of Interest

The authors declare that the research was conducted in the absence of any commercial or financial relationships that could be construed as a potential conflict of interest.

## Publisher's Note

All claims expressed in this article are solely those of the authors and do not necessarily represent those of their affiliated organizations, or those of the publisher, the editors and the reviewers. Any product that may be evaluated in this article, or claim that may be made by its manufacturer, is not guaranteed or endorsed by the publisher.

## Footnotes

^1^This also applies to pseudo-transitive verbs, which also allow two arguments and comprise the verb type used in the present study.
