# Supplementary material for: Mapping Verb Retrieval With nTMS: The Role of Transitivity
Source: Front Hum Neurosci. 2021 Sep 1;15:719461. doi: 10.3389/fnhum.2021.719461 (PMC8442843; doi:10.3389/fnhum.2021.719461)
Supplement: Supplementary file 1 [file Data_Sheet_1.docx]

**Table 1:** Participant characteristics of sample included in the present study. RMT: Resting Motor Threshold

| Participant | Handedness | RMT Left | RMT Right |
| --- | --- | --- | --- |
| 1 | 65 | 38 | 37 |
| 2 | 70 | 33 | 29 |
| 3 | 95 | 42 | 43 |
| 4 | 80 | 35 | 39 |
| 5 | 75 | 34 | 35 |
| 6 | 20 | 50 | 35 |
| 7 | 95 | 37 | 47 |
| 8 | 65 | 35 | 30 |
| 9 | 85 | 32 | 31 |
| 10 | 95 | 35 | 43 |
| 11 | 80 | 40 | 34 |
| 12 | 80 | 27 | 22 |
| 13 | -100 | 33 | 35 |
| 14 | 45 | 25 | 26 |
| 15 | 90 | 30 | 31 |
| 16 | 75 | 33 | 31 |
| 17 | 60 | 26 | 27 |
| 18 | 75 | 37 | 37 |
| 19 | 85 | 39 | 34 |
| 20 | 85 | 43 | 34 |
| Mean | 60 (SD = 43) | 35.2 (SD = 6) | 34 (SD = 6.1) |

**Table 2:** Test results for frequency, age of acquisition, naming agreement, image complexity, and number of baseline exclusions between transitive and intransitive items. Each row corresponds to a single participant due to images being excluded during baseline naming (see 2. Methods)

| Participant | Frequency | Age of acquisition | Naming agreement | Image complexity | Baseline exclusions |
| --- | --- | --- | --- | --- | --- |
| 1 | t = 1.293, p = 0.202 | t = -0.24932, p = 0.8047 | t = -0.63993, p = 0.5256 | t = -0.946, p = 0.349 | χ^2^ = 1.076, p = 0.299 |
| 2 | t = 1.647, p = 0.106 | t = -0.50369, p = 0.6182 | t = -0.2094, p = 0.8351 | t = -0.672, p = 0.504 | χ^2^ = 1.684, p = 0.194 |
| 3 | t = 2.026, p = 0.050 | t = -0.92664, p = 0.3621 | t = -0.49375, p = 0.6252 | t = -0.484, p = 0.631 | χ^2^ = 1.079, p = 0.298 |
| 4 | t = 1.578, p = 0.122 | t = -0.52368, p = 0.6048 | t = -0.13943, p = 0.8898 | t = -1.159, p = 0.253 | χ^2^ = 0.015, p = 0.902 |
| 5 | t = 1.368, p = 0.178 | t = -0.28517, p = 0.7777 | t = -0.31749, p = 0.7525 | t = -1.093, p = 0.280 | χ^2^ = 0.174, p = 0.676 |
| 6 | t = 0.810, p = 0.421 | t = 0.00, p = 1.0000 | t = -0.18824, p = 0.8515 | t = -1.580, p = 0.120 | χ^2^ = 3.222, p = 0.072 |
| 7 | t = 1.08, p = 0.284 | t = 0.18223, p = 0.857 | t = 0.098462, p = 0.9221 | t = -1.187, p = 0.242 | χ^2^ = 0.003, p = 0.950 |
| 8 | t = 0.410, p = 0.683 | t = -0.70362, p = 0.4872 | t = -0.43431, p = 0.6665 | t = -1.035, p = 0.306 | χ^2^ = 0.967, p = 0.325 |
| 9 | t = 1.094, p = 0.280 | t = 0.22299, p = 0.8253 | t = -0.73717, p = 0.4656 | t = -0.671, p = 0.506 | χ^2^ = 0.174, p = 0.676 |
| 10 | t = 1.335, p = 0.189 | t = 0.38384, p = 0.7045 | t = -0.60263, p = 0.5502 | t = -0.866, p = 0.392 | χ^2^ = 0.000, p = 1 |
| 11 | t = 0.779, p = 0.439 | t = -0.039724, p = 0.9686 | t = -0.38191, p = 0.7044 | t = -1.001, p = 0.322 | χ^2^ = 0.000, p = 1 |
| 12 | t = 1.205, p = 0.235 | t = -0.96955, p = 0.3413 | t = -0.10678, p = 0.9156 | t = -0.979, p = 0.333 | χ^2^ = 0.371, p = 0.542 |
| 13 | t = 0.918, p = 0.363 | t = 0.094298, p = 0.9256 | t = -0.32005, p = 0.7503 | t = -0.979, p = 0.332 | χ^2^ = 3.766, p = 0.052 |
| 14 | t = 1.064, p = 0.292 | t = -0.22366, p = 0.8246 | t = -0.23696, p = 0.8138 | t = -0.945, p = 0.349 | χ^2^ = 1.271, p = 0.259 |
| 15 | t = 1.607, p = 0.114 | t = -0.22701, p = 0.8219 | t = -0.16418, p = 0.8703 | t = -1.611, p = 0.114 | χ^2^ = 2.443, p = 0.118 |
| 16 | t = 0.345, p = 0.731 | t = 0.15137, p = 0.8807 | t = -1.1059, p = 0.2759 | t = -1.373, p = 0.176 | χ^2^ = 1.532, p = 0.215 |
| 17 | t = 1.949, p = 0.058 | t = -0.51292, p = 0.6123 | t = -0.52723, p = 0.6012 | t = -0.557, p = 0.580 | χ^2^ = 0.104, p = 0.746 |
| 18 | t = 0.981, p = 0.332 | t = 0.010182, p = 0.992 | t = -0.57462, p = 0.5691 | t = -1.352, p = 0.183 | χ^2^ = 1.093, p = 0.295 |
| 19 | t = 1.615, p = 0.113 | t = -0.25001, p = 0.8045 | t = -0.16616, p = 0.8688 | t = -1.494, p = 0.142 | χ^2^ = 0.483, p = 0.486 |
| 20 | t = 1.600, p = 0.116 | t = -0.40351, p = 0.6894 | t = -0.64619, p = 0.5217 | t = -0.681, p = 0.499 | χ^2^ = 2.866, p = 0.090 |
|  | | | |  |  |

**Table 3:** Types of errors in the right hemisphere for transitive and unergative verbs. W: Wald statistic; N: Nuisance parameter.

| **Error Type** | **Chi-square/Barnard’s result** | **Percentage of errors with transitives** | **Percentage of errors with unergatives** |
| --- | --- | --- | --- |
| Non-linguistic | χ^2^ = 0.01, p = .91 | 1.8% | 1.7% |
| Lexico-semantic | χ^2^ = 2.03, p = .15 | 4.4% | 3.5% |
| Grammatical | W = .68, N = .001, p = .53 | 0.05% | .1% |
| Sound | χ^2^ = 0.90, p = .34 | 2.7% | 2.3% |

**Table 4:** Types of errors in the right hemisphere according to each lobe. – : No errors of this type occurred; W: Wald statistic; N: Nuisance parameter.

| **Lobe** | **Non-linguistic errors** | **Lexico-semantic errors** | **Grammatical errors** | **Articulatory/Speech errors** |
| --- | --- | --- | --- | --- |
| Frontal | χ^2^ = 0.43, p = .50 | χ^2^ = 0.31, p = .57 | W = 1.02, N = 0.001, p = .35 | χ^2^ = 1.11; p = .29 |
| Temporal | W = 0.44, N = 0.003, p = .73 | W = -1.34, N = 0.004, p = .29 | – | χ^2^ = 0.13, p = .71 |
| Parietal | W = -1.71, N = 0.98, p = .09 | χ^2^ = 0.89, p = .34 | – | χ^2^ = 0.00, p = 1 |
